# Morphology Remodeling and Selective Autophagy of Intracellular Organelles during Viral Infections

**DOI:** 10.3390/ijms21103689

**Published:** 2020-05-23

**Authors:** Shanhui Ren, Chan Ding, Yingjie Sun

**Affiliations:** 1 Department of Avian Infectious Diseases, Shanghai Veterinary Research Institute. Chinese Academy of Agricultural Science, Shanghai 200241, China; laoren988@163.com; 2 Jiangsu Co-Innovation Center for Prevention and Control of Important Animal Infectious Disease and Zoonoses, Yangzhou University, Yangzhou 225009, China

**Keywords:** virus, intracellular organelles, rearrangement, remodeling, selective autophagy

## Abstract

Viruses have evolved different strategies to hijack subcellular organelles during their life cycle to produce robust infectious progeny. Successful viral reproduction requires the precise assembly of progeny virions from viral genomes, structural proteins, and membrane components. Such spatial and temporal separation of assembly reactions depends on accurate coordination among intracellular compartmentalization in multiple organelles. Here, we overview the rearrangement and morphology remodeling of virus-triggered intracellular organelles. Focus is given to the quality control of intracellular organelles, the hijacking of the modified organelle membranes by viruses, morphology remodeling for viral replication, and degradation of intracellular organelles by virus-triggered selective autophagy. Understanding the functional reprogram and morphological remodeling in the virus-organelle interplay can provide new insights into the development of broad-spectrum antiviral strategies.

## 1. Introduction

Viruses are obligate intracellular parasites that must rely on the cellular function for each stage of their life cycle [1,2]. To successfully enter a cell, enveloped viruses bind to surface-specific receptors through their transmembrane glycoproteins and subsequently activate intracellular signaling transduction to initiate entry; non-enveloped viruses bind through the capsid surface or projections from the capsid [3]. Viral penetration into the host cell is followed by genome uncoating, genome expression and replication, assembly of new virions, and their egress [4]. To maintain homeostasis, a fundamental function of the membrane-bound organelles is used as a scaffold to compartmentalize cellular trafficking and secretory signaling. Upon viral infection, the membranes of the intracellular organelles are remodeled and utilized by viruses as platforms to coordinate the accumulation of viral and cellular components required for efficient replication [4,5].

In addition to the rearrangement of intracellular organelles, massive viral infection also leads to the accumulation of damaged organelles, misfolded proteins, and other macromolecules. Autophagy is a conserved catabolic multistep process that non-selectively or selectively delivers large cytoplasmic proteins, including damaged organelles, into specific double-membrane autophagosome vesicles, and shuttles to the vacuole/lysosomes for degradation and recycling [6]. The process of autophagic regulation is divided into several steps: initiation, elongation, fusion, and degradation [7]. The specific targeting of cytoplasmic substrates for degradation through autophagosome depends mainly on specific cargo receptors, which contain an LC3-interacting region (LIR) motif and ubiquitin binding domain [8]. To date, several adaptor proteins, including p62/SQSTM1 [9,10,11,12], AMBRA1 [13], NBR1 [14], optineurin/OTPN [15,16], TAXIBP1 [17], CALCOCO2/NDP52 [18], BNIP3L/NIX [19,20], and BNIP3 [21], PHB2 [22], FUNDC1 [23], Cardiolipin [24], and FAM134B [25], have been identified as being involved in the recognition of cargo substrates for degradation. Most viruses activate and utilize the autophagic machinery for infectious progeny with notable exceptions, such as sindbis virus (SIV) [26,27], herpesviruses (α-, β-, and γ-) [28,29,30], human parainfluenza virus typ3 (HPIV3) [31], and human immunodeficiency virus type 1 (HIV-1) [32]. During viral infection, based on degradation substrates such as the mitochondria, peroxisome, endoplasmic reticulum (ER), lysosome, and nucleus, the selective autophagy of organelles is called mitophagy, pexophagy, ER-phagy, lysophagy, and nucleophagy, respectively [33,34].

Given the importance of membrane biogenesis in the interplay between the virus and the organelle, in this review, we briefly summarize our current knowledge about viruses’ modification of membranes morphology and biogenesis of intercellular organelles to support viral infection progeny. Moreover, we describe the potential roles of selective autophagy in the regulation of intracellular organelles upon viral infection.

## 2. Rearrangement of Intracellular Organelles during Viral Infections

To maximize their viral replication and evade host antiviral responses, viruses have evolved a plethora of strategies to hijack cellular organelles [1,35,36]. Each step of viral replication is closely accompanied by the rearrangement of intracellular organelles.

### 2.1. Remodeling of the Mitochondria for Viral Replication

**Figure 1:**
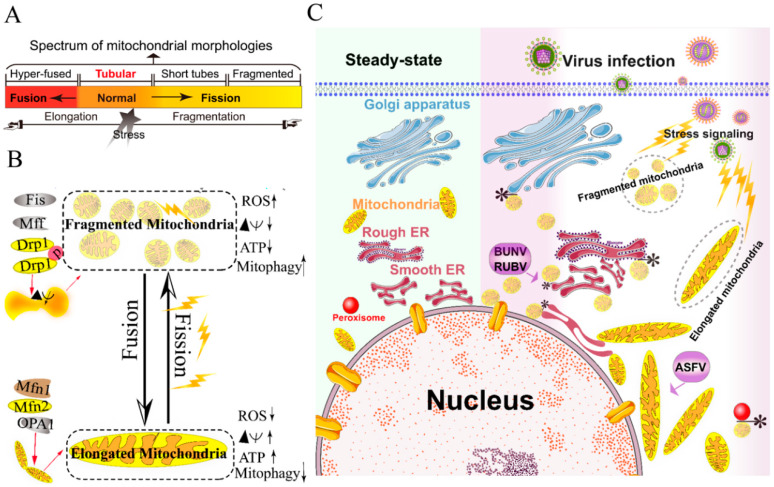
Morphology remodeling and spatial redistribution of mitochondria triggered by virus infections. (**A**) The morphological diagram of the mitochondria. Mitochondria form a dynamic network pool, which constantly undergoes rearrangement and turnover. The equilibrium regulation of mitochondrial fusion–fission is essential to maintain the integrity of mitochondria [59]. The morphology of mitochondria was divided into hyper-fused (elongated), tubular (normal), short tubes, and fragmented [50]. (**B**) Regulation of mitochondrial fusion and fission. Mitochondrial fusion is mediated by mitofusin GTPases MFN1 and MFN2 at the outer mitochondrial membrane (OMM), and OPA1 at the inner mitochondrial membrane (IMM). Mitochondrial fission is driven by the fission machinery complex, which consists of DRP-1, Fis1, and MFF. Mitochondrial hyper-fusion is a pro-survival type, which can increase the ATP production and membrane potential (Δψm), and decrease reactive oxygen species (ROS) and mitophagy [50,59]. (**C**) Proposed model for the nuclear aggregation of mitochondria and the possible interplay among intracellular organelles in response to virus infections. * symbol indicates the possible interaction site. Representative virus that increases the interactions among intracellular organelles is shown with purple rectangle. African swine fever virus, ASFV; Rubella virus, RUBV; Bunyamwera virus, BUNV.

The mitochondria are highly dynamic organelles and form interconnected tubular networks, undergoing a balance between fusion and fission in response to intracellular and/or extracellular stresses [37] (Figure 1A,B). Mitochondrial fusion involves two sets of key GTPase proteins in mammals: the mitofusin GTPases (Mfns) (Mfn1 and Mfn2) of the outer mitochondrial membrane (OMM) and optic atrophy 1 (OPA1) of the inner mitochondrial membrane (IMM) [38,39,40,41,42]. The Mfns mediate OMM fusion and cristae integrity [43]. However, the OPA1 mediates IMM fusion and cristae integrity by regulating of the mRNA splicing forms, membrane potential, and the adenosine triphosphate (ATP)-dependent diverse cellular proteases [39,40,41]. Subsequently, OMM fusions are followed by IMM fusion processes, resulting in the concomitant mixing of the mitochondrial contents and merging of two individual mitochondria. In a previous study, Cipolat et al. identified that OPA1 specific functional cross-talk with Mfn1 rather than Mfn2 is involved in the mitochondrial fusion of OMM [44]. Mitochondrial fission is a complex process that includes two distinct steps: an initial constriction of mitochondrial membranes and membrane scission. The initial constriction step narrows the mitochondrial tube diameter at the ER-mitochondria intersection zones where ER tubules wrap around the OMM. Manor et al. suggested that actin-nucleating protein spire 1C localizes to the mitochondria, directly links the mitochondria to the actin cytoskeleton and the ER, and finally promotes actin polymerization at the ER-mitochondria intersections [45]. The membrane scission of the mitochondria is primarily regulated by dynamic relative GTPase protein (DRP-1) [46]. The mitochondrial localization of DRP-1 is a cytosolic factor promoting mitochondrial fission, which powers the constriction and division of the mitochondria primarily through post-translational modification (e.g., phosphorylation) (reviewed by Lee et al. [47]). Recent studies have reported that the recruitment of DRP-1 in mammalian cells requires several accessory proteins, such as the mitochondrial fission protein 1 (Fis-1) and mitochondrial fission factor (Mff) [48]. Although such proteins are proposed to constitute the fission complex of the mitochondria, mediating mitochondrial fission using this complex has remained unclear.

Viruses have evolved several strategies to remodel the mitochondria for viral replication and assembly, including spatial distribution, morphology remodeling, and metabolism reprogramming. To maximize the effectiveness of DNA replication, African swine fever virus (ASFV) infection recruits the mitochondria around the viral factories, associated with the morphology change and accumulation of the mitochondria. It was speculated that the translation and ATP synthesis are coupled and compartmentalized around viral factories to promote virus replication [49] (Figure 1C). Normal mitochondria are dynamic organelles, and form interconnected tubular networks [37,50] (Figure 1A). The cristae remodeling of the IMM determines the assembly and stability of respiratory chain supercomplexes and respiratory efficiency [51]. In general, the mitochondrial elongation process is associated with the dimerization and activation of the ATPase function to produce additional energy [50,52]. NDV induces the hyper-fusion of the mitochondria in infected A549 cells (unpublished data), which is similar to the characteristic of Dengue virus (DENV) [53] and severe acute respiratory syndrome-coronavirus (SARS-CoVs) [54]. Notably, except for vaccinia virus (VV) [55], most viruses exploit aerobic glycolysis of the mitochondria for the production of viral progeny [36].

Moreover, viral infections may increase the inter-organellar interactions of the mitochondria with other organelles for replication. Rubella virus (RUBV) [56] and Bunyamwera virus (BUNV) [57] infections increase the membrane interactions among mitochondria, ER, and Golgi (Figure 1C), which is consistent with that of the ER-mitochondria contract that serves as a platform for inter-organellar communication [58].

To date, several reports have argued the role of Mfns in innate immunity [60,61,62]. The interaction of Mfns with the adaptor mitochondrial antiviral signaling protein (MAVS) (also called IPS-1, Cardif, or VISA) at the mitochondrial associated membrane (MAM) leads the initiation of the IFN signaling pathway [63,64]. Meanwhile, MAVS was also reported to interact with MFN2, which leads to the inhibition of inflammatory cytokine production, suggesting the MAM plays a complex role in the regulation of innate immunity [61] (detailed in review [64]). Castanier et al. also identified the cross-modulation relationship between mitochondrial dynamic and retinoic acid-inducible gene I protein (RIG-I) like receptor (RLR) signaling activation [60]. Certain viruses, such as influenza A virus (IAV) [65], measles virus (MV) [66], hepatitis B virus (HBV) [67,68,69,70], and hepatitis C virus (HCV) [67], induce selective autophagy to degrade fragmented mitochondria and evade innate immunity. Meanwhile, the non-structural (NS) protein 4B of DENV induces mitochondrial elongation via inactivation of DRP-1 and dampens the activation of RLR signal pathway to promote replication [53]. Similarly, the open reading frame-9b (ORF-9b) encoded by SARS-CoVs causes mitochondrial elongation via triggering DRP-1 degradation, and inhibits RLR signaling [54].

Collectively, viruses have evolved several strategies to hijack the mitochondria for viral genome replication and assembly, including the remodeling of mitochondrial morphology and distribution, the regulation of the fusion–fission machinery complex, and the synthesis of ATP production.

### 2.2. Rearrangement of ER and Unfolded Protein Response (UPR) during Viral Infection

**Figure 2:**
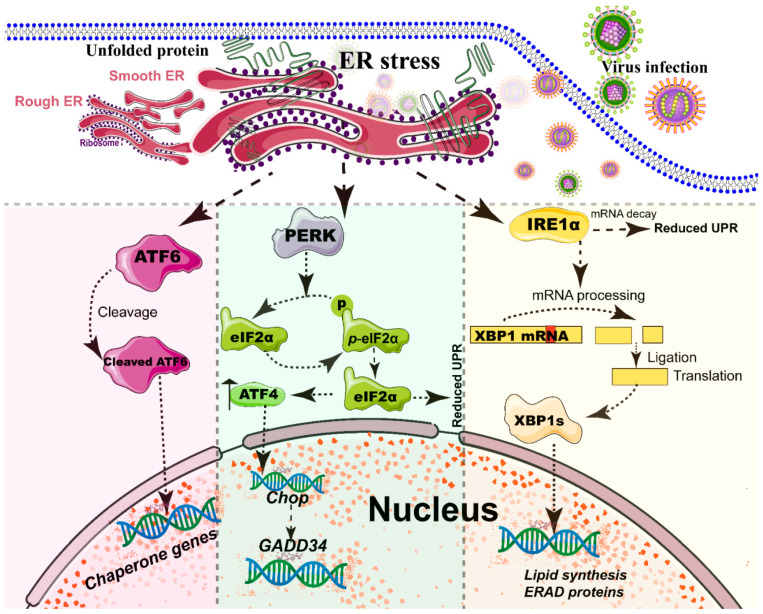
Simplified diagram of the core element of the three unfolded protein response (UPR) signaling branches of the endoplasmic reticulum (ER). During different viral infections, the ER stress activates the three stress sensor proteins: IRE1α, ATF6, and PERK (detailed in reviews [77,96]). Each sensor uses a distinct mechanism of signal transduction to drive the transcription of UPR target genes and eventually work as feedback loops to mitigate the ER stress [77,96]. Upon ER stresses, ATF6, a transcriptional factor, translocate into the Golgi compartment, where it is cleaved by the site (1/2) protease. The N-terminal cytosolic domain of cleaved ATF6 is released into cytosol and then translocated into the nucleus where it binds to ER stress-response elements to activate target genes, including XBP-1 and C/EBP-homologous protein (CHOP) [76]. The activation of PERK inhibits general protein translation by the phosphorylation of eIF2α, enabling dedicated translation of transcripts, including ATF4, a key transducer. The IRE1 branch is regulated by non-conventional mRNA splicing [77,96]. Subsequently, the activated IRE1 processes XBP1 mRNA to generate the spliced form of XBP1 protein (XBP1s), which participates in the IRE1α-mediated UPR pathway in response to ER stresses [77,96]. Eventually, the activation of the cleaved ATF6 (N-ATF6), ATF4, and XBP1 transcription factors increases the protein-folding capacity in the ER lumen. Meanwhile, IRE1 and PERK sensors also decrease the load of proteins entering the ER [77,96].

The ER, a single continuous membrane, consists of two primary structural subdomains: the nuclear envelope and the peripheral ER (a polygonal network) [71]. The nuclear envelope of ER consists of two flat membrane bilayers; the peripheral ER is composed of membrane cisternae and dynamic interconnected tubules [71,72]. The ER is the largest intracellular endomembrane system and has multiple complex functions, including Ca^2+^ storage, fatty acid synthesis, ion homeostasis, and, in particular, the quality control of newly synthesized proteins [73]. The accumulation of misfolded or unfolded proteins in the ER lumen is known as ER stress [74]. UPR and ER-associated degradation (ERAD) signaling are central to maintain the quality control of the ER [74,75]. The UPR is a signaling cascade aimed at eliminating misfolding proteins and increasing folding capacity in lumen [74]. The protein-folding conditions in the ER lumen is primarily sensed by three integrated signaling transducers: activating transcription factor 6 (ATF6) [76], double-stranded RNA-activated protein kinase-like kinase (PERK), and inositol requiring enzyme 1α (IRE1α) [58,77] (Figure 2). Each branch uses a distinct mechanism to drive the transcription of UPR signal transduction, such as ATF6 by regulated proteolysis, PERK by translational control, and IRE1 by non-conventional mRNA splicing [77]. By contrast, ERAD recognizes misfolded proteins and retro-translocates such proteins into the cytoplasm for degradation by the ubiquitin-proteasome-dependent ERAD and the autophagy-lysosome dependent ERAD [75,78].

**Figure 3:**
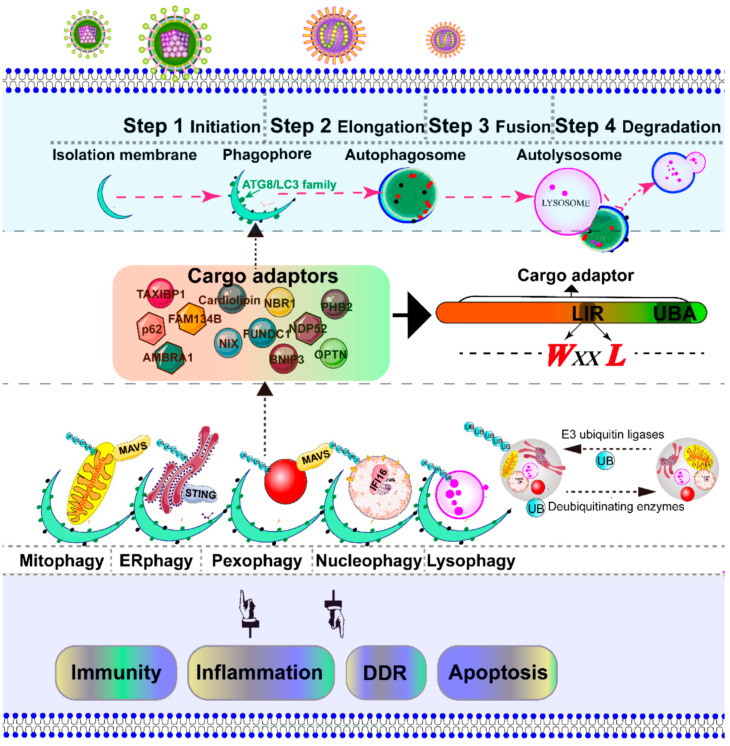
Schematic diagram of the selective autophagy. Autophagy is a conserved catabolic process, which is artificially divided into several steps: initiation, elongation, fusion, and degradation [7]. The initiation of autophagy includes the formation of the phagophore, the initial sequestering compartment. The isolation membrane elongates and expands into a double-membrane structure called an autophagosome, which chooses its cargo (the damaged organelles indicated in this figure). Completion of the autophagosomes is followed by fusion with lysosomes to form autolysosomes, where the degradation of the cargo occurs [33]. The cargo adaptor interacts directly with the damaged intracellular organelles and an autophagy modifier of the ATG8/LC3 family, which functions as a bridge between polyubiquitinated cargo and autophagosome. The autophagy adaptors contain at least an LC3-interacting region (LIR) motif and a C-terminal ubiquitin-associated domain, which is responsible for binding to mono- and poly-ubiquitinated substrates. The selective autophagic organelles are often marked and dissipated for degradation by the addition of ubiquitin by E3 ubiquitin ligases and deubiquitinating enzymes. The adaptor mitochondrial antiviral signaling protein (MAVS) is located in both mitochondria and peroxisome [159], an important downstream adapter of RIG-I mediated antiviral signaling. The stimulator of interferon genes (STING) is located in the ER, an important downstream effector of the cGAS–STING pathway [94].

A series of studies has reported that viral infections reshape the morphology and membrane remodeling of ER [1,71], and exploit various strategies to hijack the three branches of UPR for viral replication (Figure 2 and Table 1). The possible explanations were summarized as follows: first, the large malleable surface area of ER is used as a physical scaffold to protect viral RNA from degradation by cellular mRNA decay machinery [73,79]. RNA viruses have evolved several strategies to avoid the cellular mRNA decay machinery [79]. Second, viruses, particularly most RNA viruses, remodel the ER membrane to form a variety of structures for infectious progeny [5], including single-membrane spherule vesicles, double-membrane vesicles, convoluted membranes, and single-membrane sheets in the ER lumen [71]. Tenorio et al. identified that δNS and μNS of reovirus caused tubulation and fragmentation of the ER, respectively, to re-build replication sites [80], indicating that viral proteins play different roles in the rearrangement of ER membranes. Similarly, the NS4A of DENV induces the membrane arrangement of ER lumen in a 2K-regulated manner [81]. Third, viruses recruit the ER membranes into the replication and assembly compartments. The viral cytoplasmic replication site of VV [82,83], equine arteritis virus (EAV) [84], and polivirus (PV) [85] is derived directly from the ER membrane. Moreover, ASFV structural protein *p*54 plays an important role in the recruitment and transformation of the ER membranes into the envelope precursors [86]. Fourth, viruses increase the capacity and spatial rearrangement to increase ER biogenesis, including membrane protein synthesis, fatty acid change, and Ca^2+^ storage [73]. For enveloped viruses, the key molecular chaperone of ER, including Bip/GRP78 and calnexin/calreticulin, assists the folding of the extracellular domains of viral membrane glycoproteins, such as GP2a of PRRSV [87], and hemagglutinin-neuraminidase (HN) and fusion (F) proteins of NDV [88], when they translocate into the lumen of the ER. Meanwhile, the reprograming of ER biogenesis, such as Ca^2+^ storage, is required for viral replication, including HCV [89] and ASFV [90]. Fifth, viruses co-opt or subvert the ERAD processes to re-establish ER homeostasis, which actively exports the malformed proteins from the ER for degradation. Human cytomegalovirus (HCMV) [91] and IAV [92] exploit the ERAD pathway to benefit viral replication. Finally, the membrane remodeling of ER may suppress the activation of host immunity. Upon viral infections, particularly DNA viruses, stimulator of interferon genes (STING), an activated ER adaptor of the cyclic GMP-AMP synthase (cGAS)-STING signaling pathway, translocates from the ER to the ER-Golgi-intermediate compartment (ERGIC) and the Golgi apparatus, and then activates downstream molecules [93,94,95]. Therefore, we speculate that the morphology remodeling and membrane modification of ER induced by viruses may be involved in the regulation of STING trafficking, EARD degradation, and post-translational modification, and eventually evade the activation of cGAS-STING pathway (Figure 3).

### 2.3. Rearrangement of Peroxisome for Infectious Progeny

The peroxisomes are single membrane-bounded organelles that function in numerous metabolic pathways, including β-oxidation of long-chain fatty acids, detoxification of hydrogen peroxide, and synthesis of ether phospholipids and bile acids [113,114]. Notably, the mitochondria and peroxisomes share common functions in the β-oxidation of fatty acids and the reduction of damaging peroxides. Proliferation of peroxisome is largely mediated by growth and division. Peroxisomal division in mammalian cells comprises multiple processes, including membrane deformation, elongation, constriction, and fission [115]. With the exception of peroxin (PEX)-11, the peroxisomes and mitochondria share common fission machinery, including DRP-1, Mff, and Fis1 [116,117]. The fission machinery of peroxisome is orchestrated by PEX-11β and mitochondrial fission factors [115]. Mitochondrial-derived vesicles (MDVs) are involved in the transportation of mitochondrial-anchored protein ligase (MAPL), a mitochondrial outer membrane, to peroxisomes [118]. The retromer complex containing vacuolar protein sorting (Vps) 5, Vps 26, and Vps 29, a known component of vesicle transport from the endosome to the Golgi apparatus, also regulates the transport of MAPL as a binding partner from the mitochondria to peroxisomes [119].

Viruses regulate the morphology and biogenesis of peroxisomes to promote progeny replication [35]. For instance, the C-terminal of the rotavirus VP4 protein is directly located in peroxisomes via its conserved peroxisomal targeting signal [120]. Meanwhile, viruses have exploited the myristoyl-CoA produced by peroxisome biogenesis for the N-myristoylation of viral proteins [35], such as ASFV [121], indicating that peroxisomal lipid metabolism contributes to viral replication. Another typical example is the tomato bushy stunt virus (TBSV), a member of the *Tombusviridae* family, which infects a variety of plant species. McCartney et al. reported that TBSV induced the rearrangement of peroxisomes and caused vesiculation of the peroxisomal membrane, where it provided a scaffold for virus anchoring and server as the sites of viral RNA synthesis [122]. In the absence of peroxisomes, TBSV also exploits the surface of the ER membrane as a viral factory for viral replication and assembly [123]. It is suggestive of the remarkable flexibility of the virus to use host membranes for replication.

### 2.4. Hijacking of Golgi Apparatus for Infectious Progeny

The Golgi apparatus is a highly dynamic organelle whose function primarily includes saccule formation and utilization of such saccules in vesicle formation at the opposite face for delivery [124]. The normal cellular secretory pathway, bidirectional transport between the ER and Golgi apparatus, is mediated by tubulovesicular transport containers that depend on two coat protein complexes, COP-I and COP-II. COP-II establishes a membrane flow from the ER to the Golgi complex [125]. However, COP-I coat acts in retrograde transport from the Golgi back to the ER [126].

In general, certain viruses utilize the cellular trafficking and secretory pathway of the ER-Golgi transport system to replicate/release their progeny [1]. PV utilizes the components of the ADP-ribosylation factor (Arf) family of small GTPases [127] and cellular COP-II proteins [128] to form membrane-bound replication complex for viral replication, indicating that PV hijacks the components of the cellular secretory pathway for replication. As shown in Table 2, metonaviridae [56] and peribunyaviridae [57] hijack the Golgi complex to re-construct it as a viral factory for viral replication. For example, RUBV [56] and BUNV [57] infections modify cell structural morphology and remodel the Golgi complex as a viral factory during the entire life cycle. Furthermore, host secretory signaling is also crucial for innate and acquired immune responses, such as the exportation of proinflammatory and antiviral cytokines. Nearly 25 years ago, Doedens et al. reported that the 2B and 3A proteins of PV inhibited cellular protein secretion by directly blocking the transportation from the ER to the Golgi apparatus [129], indicating that the functional secretory protein is not indispensable for viral RNA replication. Mechanistically, Dodd et al. identified that the inhibition of 3A protein of PV on the ER to Golgi limited the antiviral cytokine secretion of native immune response and inflammation, including interleukin-6, interleukin-8, and β-interferon [130]. Deitz et al. also identified that PV 3A protein reduced the presentation of new antigens on the cell surface [131]. Considering that the ER adaptor STING was also located on the Golgi and ERGIC [93], we hypothesized that the membrane remodeling and modification of Golgi induced by viruses might also be involved in the regulation of cGAS-STING pathways (Figure 3). Collectively, all these data suggest that enteroviruses, such as PV and CVB, have evolved a series of strategies to hijack cellular trafficking and secretion for viral replication.

### 2.5. Role of the Lysosome and Endosome in Viral Infections

The lysosome, an acidic and membrane-bound organelle, acts as a cellular recycling center and is filled with a number of hydrolases [152]. The lysosome-based degradation processes are subject to reciprocal regulation [153]. Lysosomes degrade unwanted materials that are delivered either from outside via the endocytic pathway or from inside via the autophagic pathway [153,154]. For viral replication and assembly, certain viruses, including *Alphaviruses* [146], such as semliki forest virus (SFV) [145], exploit the membrane surface of the endosome and lysosome as a viral factory. Similarly, RUBV also can modify the membrane of lysosomes for a viral factory [142]. Meanwhile, the Toll-like receptors (TLR), such as TLR 3/7/9, are located on the endosome, indicating that the endosome also plays an important role in innate immunity [94]. Therefore, we speculate that another possible strategy is that viruses, particularly DNA viruses, evade the TLR-mediated activation of the NF-κB and transcription of proinflammatory cytokines. HBV infection suppresses TLR-9 expression and prevents TLR9 promoter activity in human primary B cells [155]. Interestingly, DENV, a positive-stand RNA virus, activates the TLR9 by inducing mtDNA release in human dendritic cells [156]. Additionally, the endosomal-lysosomal sorting system is a complex and dynamic vesicular sorting signaling, which is fundamental to maintain homeostasis [157]. Viruses, particularly enveloped viruses such as HIV [151], have evolved several strategies to hijack the endosomal sorting complex required for the transport (ESCRT) complex to facilitate viral budding. Collectively, all these data indicate that different viruses utilize different strategies to hijack the endosome/lysosome for viral progeny.

## 3. Degradation of Intracellular Organelles by Virus-Triggered Selective Autophagy

Macroautophagy was initially described as a non-selective degradation process [33]. However, selective autophagy is characterized as a highly regulated and specific degradation pathway targeting damaged organelles [158]. The initiation of autophagy includes the formation of the phagophore from membrane precursors. The phagophore elongates by the ubiquitin-like conjugation systems and LC3-II-phosphatidylethanolamine to form the autophagosome [33]. The autophagosome sequesters within damaged intracellular organelles, such as the mitochondria, ER, peroxisome, nucleus, and lysosome, and undergoes fusion with a lysosome to form an autolysosome, where degradation occurs [33]. Depending on the targeted organelles, selective autophagy can be divided into mitophagy, pexophagy, ER-phage, lysophagy, nucleophagy, etc. [33] (Figure 3). In most cases, viral infection leads to the severe damage of intracellular organelles, which subsequently initiates selective autophagy to degrade these damaged organelles. Therefore, selective autophagy is the protective mechanism for cells to maintain cell homeostasis. In contrast, in some cases, selective autophagy could be utilized by a virus to promote their replication. Here, we address the principal mechanism of selective autophagy triggered by viral infection, with an emphasis on mitophagy and pexophagy, which has been best described to date.

### 3.1. Overview of Mitophagy and Pexophagy Signaling

The selective elimination of damaged mitochondria is termed mitophagy and is a type of macroautophagy [6] (Figure 3). The fragmented mitochondria are easier to recognize by autophagosome than the elongated mitochondria because of imbalanced fission–fusion of the mitochondria [160,161,162,163]. The canonical ubiquitin-dependent PTEN-induced putative kinase protein 1(PINK1)-Parkin mitophagy signal has been validated in multiple model systems by different approaches [164,165,166]. Mitochondrial PINK1 is rapidly turned over on the bioenergetically well-coupled mitochondria by proteolysis by presenilin-associated rhomboid like protein (PARL) [167], but PINK1 is selectively stabilized on the mitochondria with loss of membrane potential (Δ_ψ_m) [168,169]. Selective accumulation of PINK1 recruits downstream Parkin, a cytosolic RING-between-RING E3 ligase, to the impaired mitochondria [169]. In turn, Parkin-induced mitophagy is strictly dependent on depolarization-induced accumulation [167,169]. Chen et al. previously reported that PINK1-phosphorylated Mfn2 as a receptor-mediated Parkin recruitment to the damaged mitochondria [166]. Meanwhile, the phosphorylated-ubiquitin on ser 65 (*p-*Ser65-Ub) chains is also identified as a potent Parkin activator and receptor, which leads to the onset of mitophagy [170,171,172]. Notably, Lazarou et al. recently reported that PINK1 recruits NDP52 and OPTN cargo receptors, but not p62/SQSTM1, to directly activate mitophagy, independently of Parkin [173]. Similarly, to protect against ischemic brain injury, BNIP3L/NIX-mediated mitophagy is independent of Parkin [19,20]. Furthermore, upon mitophagy induction, AMBRA1 binds to the autophagosome adapter LC3 to initiate a powerful mitophagy, promoting canonical Parkin PINK1-dependent and Parkin-independent mitochondrial clearance [13]. All the aforementioned data show that Parkin may act as an amplifier, which is not indispensable for mitophagy. Intriguingly, the protein kinase PINK1 and Parkin are also involved in the generation of MDVs [174].

Peroxisome homeostasis is regulated by division and pexophagic degradation. The degradation of the aberrant peroxisomes by selective autophagy is known as pexophagy [33,114,175] (Figure 3). Four different types of pexophagy are identified in mammalian cells [115], including ubiquitin-mediated pexophagy [176], NBR1-induced pexophagy [177], PEX3-induced pexophagy [178], and PEX14-LC3 interaction-mediated pexophagy [179]. Compared with pexophagy in yeast [114], the underlying mechanisms of pexophagy in mammalian cells are less elucidated. The ubiquitination of mammalian PEX5 [180,181] and PMP70 [181] has been identified in pexophagy. In response to reaction oxygen species, ataxia-telangiectasia-mutated kinase phosphorylates PEX5 and eventually links the peroxisome with the adaptor p62/SQSTM1 for pexophagy [180]. During amino acid starvation, the peroxisomal E3 ubiquitin ligase PEX-2 ubiquitinates downstream PEX5 and PMP70 and subsequently degrades peroxisome in an NBR1-dependent manner [181].

Under various stresses, complex signaling pathways are involved in the activation and regulation of mitophagy and pexophagy. The detailed mechanism needs to be further elucidated in future research.

### 3.2. Virus Triggers Mitophagy and Pexophagy to Suppress MAVS-Dependent RLR Signaling

The RLR-dependent type I interferon responses are regulated by MAVSs, which are initially thought to be only located in the OMM of the mitochondria [182]. Upon viral infection, MAVSs bind to RLRs and promote the activation of downstream signal transduction [93,94]. Khan et al. reported that HCV attenuated the innate immunity via Parkin-dependent recruitment linear ubiquitin assembly complex to the mitochondria [183]. Similarly, Edmonston strain (MV-Edm), an attenuated MV, triggered MAVS degradation via p62/SQSTM1-mediated mitophagy to weaken the RLR signal [66]. The matrix protein (M) of HPIV3 [184] and PB1-F2 of IAV [185] induce the mitophagy Parkin-PINK1-independent pathway to suppress innate immunity. More importantly, Dixit et al. recently identified that partial MAVSs are also located on the peroxisome for antiviral signal transduction [159]. Upon viral infection, the mitochondria and peroxisomes are not just simple metabolic organelles, but rather serve as a critical subcellular platform for antiviral immunity, which expands our understanding about the integration of antiviral networks of the intracellular organelles. Mitochondrial MAVSs may mediate the proapoptotic signaling of innate immune activity against viral infections [186,187]. Previous reports suggested that HCV proteolytically cleaved the MAVS from the mitochondria by NS3/4A [188,189,190] at cysteine 508 amino residue rather than degrading the MAVS to cripple innate immunity [191]. Horner et al. further identified that NS3/4A of HCV targeted the mitochondrial-associated membrane (MAM) and cleaved MAVSs from the MAM, but not from the mitochondria to ablate the immune actions of the MAVS signalosome during HCV infection [192]. Taken together, various viruses have evolved a plethora of strategies to exploit mitophagy and pexophagy to suppress MAVS-dependent RLR signaling to maximize their own replication.

### 3.3. ER-Phagy (reticulophagy) Trigged by Viral Infection

Different viruses have exploited different strategies to utilize the UPR of the ER for viral replication (Table 1). In our laboratory, we found that NDV activated all three branches of the UPR of the ER to facilitate NDV replication [105]. Synergistic expression of HN and F of virulent NDV is necessary for the UPR activation of the ER [88]. However, the exact mechanism of how a virus leads to the accumulation of unfolded proteins in the lumen and utilizes the UPR of the ER needs to be further investigated. The selective degradation of ER is termed ER-phagy [193,194]. Upon the stimulation of an ER stress inducer, the signaling networks of ATF6, PERK, and IRE1α, and cellular Ca^2+^, are necessary for the activation of ER-phagy at different stages, including induction, vesicle nucleation, and elongation [194] (Figure 3). PERK and IRE1α branches of ER stress are indispensable for DENV-induced autophagy [98]. To date, FAM134 [25], BNIP3/Nix [21], and p62/SQSTM1 [12] have been identified as cargo receptors of ER-phagy. Additionally, Tomar et al. [195] reported that TRIM13, an ER-resident ubiquitin E3 ligase, regulates the ER-phagy process and ER stress. Considering the important role of the ER-localized STING in anti-viral innate immunity [93,95], we speculate that ER-phagy may be involved in the inhibition of the cGAS-STING pathway during virus infections, particularly DNA virus infection (Figure 3). The precise underlying mechanisms of ER-phagy upon viral infection need to be further investigated.

### 3.4. Lysophagy and Nucleophagy Trigged by Viral Infection

The selective autophagy is initiated by isolation membranes. Subsequently, the isolation membranes are close to one another to form double membrane bound autophagosomes, and eventually fuse with lysosomes for degradation [6,34]. Notably, the autophagosome does not have the ability to degrade its contents. Only after fusion with the lysosome, which provides an acidic environment and hydrolases, can the autophagosome degrade the autophagosomal contents. Numerous inducers can trigger lysosomal membrane permeabilization and the consequent leakage of the lysosomal content into the cytosol, which eventually leads to so-called “lysosomal cell death” [152]. The removal of damaged lysosomes by selected autophagy is termed lysophagy [33,34] (Figure 3). Moreover, nucleophagy is a selective autophagy, which selectively removes damaged or non-essential material by the autophagy pathway [113,196] (Figure 3). Recently, Unterholznoer et al. have identified IFI16, a PYHIN protein, as a new DNA sensor [197]. Considering the nuclear distribution of IFI16, we speculate that nucleophagy may involve the IFI16-dependent innate immunity (Figure 3). Compared with mitophagy and pexophagy, many questions regarding the molecular details of lysophagy and nucleophagy pathways should be further elucidated. One of the interesting questions is how the nucleus and lysosome are sequestered by the phagophore and recognized by the cargo adaptor in response to viral infections.

## 4. Concluding Remarks and Future Perspectives

In the current review, we present a brief overview of the quality control strategies of intracellular organelles in mammalian cells in response to viral infection. Although distinct steps of the viral life cycle have long been known to associate with the abundant membrane rearrangement of intracellular organelles [4], a detailed understanding of the interplay of virus and host, particularly the interaction between individual viral protein and organelle component, has remained unclear. Several important scientific questions remain unelucidated. First, what is the mechanism to coerce the host translational machinery into synthesizing viral proteins in the face of ongoing infectious progeny? Second, how do viral and cellular proteins contribute to the re-construction of viral replication factories in different subcellular membranes sites? Third, what are the viral proteins and cellular factors that are required for membrane remodeling and that metabolize reprogramming in virus-infected cells?

Moreover, although we have made great progress in the understanding of selective autophagy, the assembly site of a double membrane has remained unclear. The assembly of the phagophore may require various membranes, including the ER [198], ER-Golgi intermediate compartments (ERGIC) [199], ER-mitochondria junctions [200], mitochondria [201], mitochondrial-derived vesicles (MDVs) [202], Golgi-endosomal membranes [203], and the plasma membrane [204]. Upon DNA or RNA viral infection, further work is needed to decipher the exact phagophore assembly site of selection autophagy during viral infections. More importantly it remains unclear how the host cell initiates the “eat-me” signal for the elimination and the elongation of the phagophore membrane around targeted organelles. Every selective autophagy pathway requires a specific cargo receptor, which bridges ubiquitinated organelles to LC3/Atg8 family membranes to link with the autophagy machinery. In mammalian cells, several cargo proteins, including p62/SQSTM1, CALCOCO2/NDP52, NBR1, optineurin/OPTN, AMBRA1, BNIP3L/NIX, BNIP3, FUNDC1, TAXIBP1, cardiolipin, prohibitin-2/PHB-2, and FAM134B (Table 3), have been identified; however, the exact processes of recognition and specific selection of damaged organelles for degradation by selective autophagosomes during viral infection remain poorly understood. Notably, Lazarou et al. identified that NDP52 and OPTN are the primary receptors for PINK1-dependent mitophagy, independent of Parkin. PINK1-generated phospho-ubiquitin directly serves as the “eat-me” signal on the mitochondria [173], which extends our understanding of classical PINK1-Parkin mitophagy signaling. Furthermore, Tank-binding kinase 1 is involved in the phosphorylation of cargo receptors, including OPTN and NDP52, to create an “eat-me” signal at the autophagy-relevant site [15]. The difference between the autophagy receptor NDP52 and p62 determines the species-specific impact of the autophagy machinery during CHIKV infection, indicating that a receptor may regulate viral infection in a species-dependent manner [205]. Recently, cardiolipin of the inner mitochondrial membrane phospholipid was found to serve as an “eat-me” signal for mitochondrial clearance from neuronal cells [24]. Meanwhile, Wei et al. [22] recently identified the prohibitin-2/PHB-2 as a specific mitophagy receptor of IMM for autophagic degradation. Interestingly, the matrix protein of HPIV [184] and the PB1-F2 protein of IAV [185] can be a receptor in the induction of mitophagy. Specific selection of cargo proteins during damaged organelle degradation may be primarily dependent on the targeted organelle and viral characteristic.

Extensive improvement of our understanding of the cross-talk between viruses and organelles must depend on the innovative applications of new techniques and materials [206], such as the single-particle tracking method, the ribopuromycylation method, single cell RNA-seq, three-dimensional (3D)-reconstruction of electron microscopy, image-based genome-wide RNA interference screens, haploid genetic screens, yeast two-hybrid screens, a modern ultra-structural technique, and, in particular, a high-throughput and genome-scale CRISPR-Cas screening technique. Electron tomography and 3D imaging technology are being successfully applied to the study of virus-cell interactions, such as for EAV [84], RUBV [56], and BUNV [57]. Similarly, Ertel et al. recently revealed new interior and exterior features of RNA replication compartments of the non-human flock house nodavirus via a cryo-electron tomography technique [144]. Based on image-based genome-wide siRNA screening, Orvedahi et al. identified that SMAD specific E3 ubiquitin protein ligase 1 (SMURF1), as a newly recognized mediator, functions in mitophagy triggered by viral infection but not in starvation-induced autophagy [207]. Researchers should keep a closer eye on advanced technological breakthroughs and combine these advanced technological breakthroughs into a comprehensive understanding of virus–organelle interaction. The discovery of the underlying virus–host molecular mechanism already has an overlapping function to multiple viruses, which advance the discovery of host druggable targets and development of broad-spectrum antiviral approaches.

## Figures and Tables

**Table 1:** Viruses activate and exploit the UPR branch of ER for viral replication.

Viruses	Family	Genome Structure	Virion Structure	Viral Protein	ATF6	PERK	IRE1α	Ref
PRRSV	*Arteriviridae*	Linear, ssRNA(+)	Enveloped; Spherical	?	×	√	√	[87]
IBV	*Coronaviridae*	Linear, ssRNA(+)	Enveloped; Spherical	?	×	×	√	[97]
DENV	*Flaviridae*	Linear, ssRNA(+)	Enveloped; Spherical	?	×	√	√	[98]
JEV	*Flaviridae*	Linear, ssRNA(+)	Enveloped; Spherical	NS4B	√	√	√	[99,100,101]
TBEV	*Flaviridae*	Linear, ssRNA(+)	Enveloped; Rounded	?	√	×	√	[102]
HCV	*Flaviridae*	Linear, ssRNA(+)	Enveloped; Spherical	?	√	×	×	[103]
IAV	*Orthomyxoviridae*	Segmented, ssRNA (-)	Enveloped; Rounded	?	×		√	[104]
NDV	*Paramyxoviridae*	Linear, ssRNA(-)	Enveloped; Spherical	F and HN	√	√	√	[88,105]
MCMV	*Herpesviridae*	Linear, ds DNA	Enveloped; Spherical	?	?	√	×	[106,107]
HSV-1	*Herpesviridae*	Linear, dsDNA	Enveloped; Spherical	UL41/ICP0/γ_1_34.5	√	√	×	[108,109,110,111]
HBV	*Hepadnaviridae*	Circular dsDNA	Enveloped; Spherical	?	√	×	√	[112]

During different viral infections, the ER stress activates the three stress sensor proteins: IRE1α, ATF6, and PERK (review in the references [77,96]). The following abbreviations are used in this table: murine cytomegalovirus, MCMV; avian coronavirus infectious bronchitis virus, IBV; porcine reproductive and respiratory syndrome virus, PRRSV; hepatitis C virus, HCV; hepatitis B virus, HBV; influenza A virus, IAV; human herpes simplex virus-1, HSV-1; dengue virus, DENV; tick-borne encephalitis virus, TBEV; Japanese encephalitis virus, JEV; Newcastle disease virus, NDV. The symbols √, ×, and ? indicate activation, inhibition, and unknown, respectively.

**Table 2:** Intracellular compartment sites for viral replication and assembly.

Family	Viruses	Genome Structure	Virion Structure	Replication Site	Ref	Assembly Site	Ref
*Asfarviridae*	ASFV	Liner, dsDNA	Enveloped, spherical	Nuclear and cytoplasmic	[49,132,133]	ER	[86,90]
*Poxviridae*	VV	Liner, dsDNA	Enveloped, brick-shaped	cytoplasmic	[134]	ERGIC, ER	[82,83]
*Arteriviridae/Arteriviruses*	EAV, PRRSV	Liner, ssRNA (+)	Enveloped, spherical	Cytoplasmic double membranes	[84,135]	ER	[84]
*Coronaviridae/Coronavirus*	SARS/MHV	Liner, ssRNA (+)	Enveloped, spherical	Cytoplasmic double membranes	[136]	ERGIC, Golgi, ER	[137,138]
*Flaviviridae/Hepacivirus*	HCV	Linear, ssRNA(+)	Enveloped, spherical	Cytoplasmic	[139]	Autophagosome	[140,141]
*Metonaviridae/Rubivirus*	RUBV	Liner, ssRNA (+)	Enveloped, spherical isometric capsid?	Cytoplasmic	[56]	Golgi, Lysosome	[56,142]
*Nodaviridae*	FHV	Linear, ssRNA(+)	Non-envelop, icosahedral	Cytoplasmic	[143,144]	OMM	[143,144]
*Togaviridae/Alphavirus*	SFV	Liner, ssRNA (+)	Enveloped, spherical and icosahedral	Cytoplasmic	[145,146]	Endosome/Lysosome	[145,146,147]
*Tombusviridae*	TBSV	Linear, ssRNA(+)	Non-envelop, icosahedral	Cytoplasmic	[123]	Peroxisome, ER	[122,123]
*Picornaviridae/Enterovirus*	CV/PV	Linear, ssRNA (+)	Non-envelop, spherical	Cytoplasmic	[148]	ER	[85,148]
*Orthomyxoviridae*	IAV	Segmented, ssRNA (−)	Enveloped; Rounded	Nuclear	[149]	ER, Golgi	[149]
*Peribunyaviridae/Bunyavirus*	*BUNV*	Segmented, ssRNA (−)	Enveloped, spherical	cytoplasmic	[57]	Golgi	[57,150]
*Retroviridae/Lentivirus*	HIV	Linear, ssRNA (+)	Enveloped, Spherical	Nuclear	[151]	ER or Golgi	[151]

The following abbreviations are used in this table: African swine fever virus, ASFV; rubella virus, RUBV; Bunyamwera virus, BUNV; semliki forest virus, SFV; Flock house virus, FHV; tomato bushy stunt virus, TBSV; severe acute respiratory syndrome, SARS; murine hepatitis virus, MHV; porcine reproductive and respiratory syndrome virus, PRRSV; equine arteritis virus, EAV; influenza A virus, IAV; human immune deficiency, HIV; vaccinia virus, VV; poliovirus, PV; coxsackieviruses, CV; reticulum-Golgi intermediate compartment, ERGIC; outer mitochondrial membranes, OMM.

**Table 3:** Cargo adaptors involved in the selective autophagy in mammals.

Name	Mitophagy		ER-Phagy		Pexophagy		Nucleophagy		Lysophagy	
Cargo Proteins	Mitochondria	Ref	ER	Ref	Peroxisome	Ref	Nucleus	Ref	Lysosome	Ref
p62/SQSTM1	√	[10]	√	[12]	√	[175,176,180]	?	-	√	[208,209]
NDP52	√	[173,210]	?	-	?	-	?	-	?	-
NBR1	√	[14]	?	-	√	[175,177,181]	?	-	?	-
OPTN	√	[15,16,173,210]	?	-	?	-	?	-	?	-
AMBRA1	√	[13]	?	-	?	-	?	-	?	-
BNIP3L/NIX	√	[19,20]	?	-	?	-	?	-	?	-
BNIP3	√	[21,211]	√	[21]	?	-	?	-	?	-
TAX1BP1	√	[15,17]	?	-	?	-	?	-	?	-
FUNDC1	√	[23]	?	-	?	-	?	-	?	-
Cardiolipin	√	[24]	?	-	?	-	?	-	?	-
Prohibitin2	√	[22]	?		?	-	?	-		-
FAM134B	?	-	√	[25]	?	-	?	-	?	-

The cargo adaptor functions as a bridge between the polyubiquitinated cargo and autophagosome, which is required for selective autophagy. The autophagy adaptors interact directly with the damaged intracellular organelles and an autophagy modifier of the ATG8/LC3 family. The symbols √, ×, and ? indicate activation, inhibition, and unknown, respectively.

## Abbreviations

ATF6	Activating transcription factor 6
ATP	Adenosine triphosphate
AMBRA1	Autophagy and beclin-1 regulator 1
ASFV	African swine fever virus
BUNV	Bunyamwera virus
BNIP3L/NIX	BCL2 interacting protein 3 like
BNIP3	BCL2 interacting protein 3
cGAS	Cyclic GMP-AMP synthase
CVB	Coxsackievirus B
CHIKV	Chikungunya virus
CALCOCO2/NDP52	Calcium binding and coiled-coil domain 2/Antigen nuclear dot 52 kDa protein
COP	Coat protein complex
CV	Coxsackieviruses
DENV	Dengue virus
DRP-1	Dynamic relative GTPase protein
ERGIC	ER-Golgi-intermediate compartment
ER	Endoplasmic reticulum
ERAD	ER-associated degradation
EAV	Equine arteritis virus
FHV	Flock house virus
FUNDC1	FUN14 domain containing 1
FAM134B	Family with sequence similarity 134, member B
HIV	Human immune deficiency
HPIV3	Human parainfluenza virus typ3
HCV	Hepatitis C virus
HSV-1	Human herpes simplex virus-1
HBV	Hepatitis B virus
IAV	Influenza A virus
IMM	Inner mitochondrial membranes
IBV	Avian coronavirus infectious bronchitis virus
IAV	Influenza A virus
IRE1α	Inositol requiring enzyme 1α
JEV	Japanese encephalitis virus
LC3	Light chain 3
LIR	LC3-interacting region
MHV	Murine hepatitis virus
Mfns	Mitofusin GTPases
Mfn1	Mitofusin 1
Mfn2	Mitofusin 2
Mff	Mitochondrial fission factor
MAM	Mitochondrial-associated-membrane
MCMV	Murine cytomegalovirus
MV	Measles virus
MAPL	Mitochondrial-anchored protein ligase
MDVs	Mitochondrial-derived vesicles
MAVS	Mitochondrial antiviral signaling protein
NDV	Newcastle disease virus
NBR1	Next to BRCA1 gene 1 protein
OMM	Out mitochondrial membranes
OPA1	Optic atrophy 1
OPTN	Optineurin
PERK	Double-stranded RNA-activated protein kinase-like kinase
PRRSV	Porcine reproductive and respiratory syndrome virus
PHB2	Prohibitin 2
PEX	Peroxin
PV	Polioviruses
PINK1	PTEN-induced putative kinase protein 1
RUBV	Rubella virus
ROS	Reactive oxygen species
Ref	Reference
RIG-I	Retinoic acid-inducible gene I protein
STING	Stimulator of interferon genes
SIV	Sindbis virus
SFV	Semliki forest virus
SARS	Severe acute respiratory syndrome
TBSV	Tomato bushy stunt virus
TBEV	Tick-borne encephalitis virus
TAXIBP1	Tax1 binding protein 1
UPR	Unfolded proteins response
UPR^mt^	Unfolded protein response (UPR) of mitochondria
